# EXCHANGE-2: investigating the efficacy of add-on plasma exchange as an adjunctive strategy against septic shock—a study protocol for a randomized, prospective, multicenter, open-label, controlled, parallel-group trial

**DOI:** 10.1186/s13063-023-07300-5

**Published:** 2023-04-15

**Authors:** Sascha David, Christian Bode, Klaus Stahl, for the EXCHANGE-2 Study group, Julius Schmidt, Benjamin Seeliger, Thorben Pape, Bernhard Schmidt, Marius M. Hoeper, Heiner Wedemeyer, Tobias Welte, Kai Schmidt-Ott, Pedro David Wendel Garcia, Daniel A. Hofmänner, Rea Andermatt, Reto Schuepbach, Andriyana Bankova, Hans-Joerg Gillmann, Thomas Stueber, Carolin Jung, Andre Gerdes, Christian Putensen, Andrea Sauer, Lennart Wild, Felix Lehmann, Markus A. Weigand, Christian Nusshag, Judith Schenz, Sebastian O. Decker, Mascha O. Fiedler, Florian Uhle, Michael Bauer, Julia Leonhardt, Frank Bloos, Silke Rummler, Philipp Enghard, Abakar Magomedov, Daniel Zickler, Julius Kunz, Jochen Dutzmann, Alexander Vogt, Matthias Girndt, Silke Markau, Mathias Kochanek, Jan-Hendrik Naendrup, Alexander Zarbock, Melanie Meersch, Thilo von Groote, Mahan Sadjadi, Carola Wempe, Steffen Mitzner, Markus Heim, Stefanie Pilge, Nicolas Bubendorfer, Gerhard Schneider, Tobias Lahmer, Sebastian Rasch, Thorsten Brenner, Marc M. Berger, Jens Brands, Florian Espeter, Julius Freytag, Stefan Kluge, Dominik Jarczak, Axel Nierhaus, Jan T. Kielstein, M. Winkler, Thomas Fühner, Jan Schmieszek, Jan Menne, Martin Sauer, Georg Richter, Ingmar Lautenschläger, David Radke, Ansgar Reising, Alexander Keil, Joern Bramstedt, Mustafa Fahham, Carsten Willam, Larissa Herbst, Karl Bihlmaier, Christoph Buettner, Peter Schellongowski, Elisabeth Lobmeyr-Längle, Gürgkan Sengölge, Thomas Staudinger, Joerg C. Schefold, Philipp Venetz, Jan Waskowski, Carmen A. Pfortmueller, Michael Joannidis, Gert Mayer, Romuald Bellmann, Armin Koch, Xiaofei Liu

**Affiliations:** 1 Institute of Intensive Care Medicine, University Hospital Zurich, Rämistrasse 100, 8091 Zurich, Switzerland; 2 Department of Nephrology and Hypertension, Hannover Medical School, Hannover, Germany; 3 Department of Anesthesiology and Intensive Care Medicine, University Hospital Bonn, Bonn, Germany; 4 Department of Gastroenterology, Hepatology and Endocrinology, Hannover Medical School, Hannover, Germany

**Keywords:** Sepsis, Septic shock, Plasmapheresis, Randomized controlled trial, Extracorporeal therapy

## Abstract

**Background:**

Sepsis is as a life-threatening organ dysfunction caused by a dysregulated host response to an infection. The mortality of sepsis and particular of septic shock is very high. Treatment mostly focuses on infection control but a specific intervention that targets the underlying pathological host response is lacking to the present time.

The investigators hypothesize that early therapeutic plasma exchange (TPE) will dampen the maladaptive host response by removing injurious mediators thereby limiting organ dysfunction and improving survival in patients with septic shock. Although small prospective studies demonstrated rapid hemodynamic stabilization under TPE, no adequately powered randomized clinical trial has investigated hard outcomes.

**Methods:**

This is a randomized, prospective, multicenter, open-label, controlled, parallel-group interventional trial to test the adjunctive effect of TPE in patients with early septic shock. Patients with a refractory (defined as norepinephrine (NE) ≥ 0.4 μg/kg/min ≥ 30 min OR NE 0.3 μg/kg/min + vasopressin) and early (shock onset < 24 h) septic shock will be included. The intervention is a standard TPE with donor fresh frozen plasma (1.2 × individual plasma volume) performed within 6 h after randomization and will be compared to a standard of care (SOC) control arm. The primary endpoint is 28 days mortality for which the power analysis revealed a group size of 137 / arm (*n* = 274) to demonstrate a benefit of 15%. The key secondary objective will be to compare the extent of organ failure indicated by mean SOFA over the first 7 days as well as organ support-free days until day 28 following randomization. Besides numerous biological secondary, safety endpoints such as incidence of bleeding, allergic reactions, transfusion associated lung injury, severe thrombocytopenia, and other severe adverse events will be assessed during the first 7 days. For exploratory scientific analyses, biomaterial will be acquired longitudinally and multiple predefined scientific subprojects are planned. This study is an investigator-initiated trial supported by the German Research Foundation (DFG, DA 1209/7–1), in which 26 different centers in Germany, Switzerland, and Austria will participate over a duration of 33 months.

**Discussion:**

This trial has substantial clinical relevance as it evaluates a promising adjunctive treatment option in refractory septic shock patients suffering from an extraordinary high mortality. A positive trial result could change the current standard of care for this septic subgroup. The results of this study will be disseminated through presentations at international congresses, workshops, and peer-reviewed publications.

**Trial registration:**

ClinicalTrials.gov NCT05726825, Registered on 14 February 2023.

## Admininistrative information

**Table:** 

**Title**	EXCHANGE-2: Investigating the efficacy of add-on plasma exchange as an adjunctive strategy against septic shock – a randomized, prospective, multicenter, open-label, controlled, parallel-group trial
**Trial Registration**	ClinicalTrials.gov, NCT05726825, Registered on 02/14/2023
**Protocol Version**	Version 1.2, January 2023
**Funding**	German Research Foundation (DFG, DA 1209/7–1) The German Research Foundation is neither involved in developing the design of the study nor in collection, analysis, and interpretation of the data or in writing of the manuscript.
**Author Details**	Sascha David^1,2^, Christian Bode^3^ and Klaus Stahl^4^ ^1^Institute of Intensive Care Medicine, University Hospital Zurich, Zurich, Switzerland ^2^Hannover Medical School, Department of Nephrology and Hypertension, Hannover, Germany ^3^University Hospital Bonn, Department of Anesthesiology and Intensive Care Medicine, Bonn, Germany ^4^Hannover Medical School, Department of Gastroenterology, Hepatology and Endocrinology
**Name and Contact Information for Trial Sponsor**	Hannover Zentrum für Klinische Studie (ZKS), Hannover Medical School Carl-Neuberg Strasse 1, 30,625 Hannover, Germany
**Role of Sponsor**	Investigator-initiated trial

## Trial status

The current version of the study protocol described in the manuscript is Version 1.2, January 2023. The trial was registered at ClinicalTrials.gov (NCT05726825, date of registration: February 14, 2023, last update: February 14, 2023). No patient has yet been recruited. Anticipated start of trial recruitment will be in June 2023. Approximate end of recruitment will be May 2025.

## Introduction

### Background and rationale

Sepsis is defined as a life-threatening organ dysfunction caused by a dysregulated host response to an infection [1]; in septic shock, profound circulatory, cellular, and metabolic abnormalities are associated with high mortality [2]. Sepsis is a major healthcare problem, affecting millions of individuals around the world each year. Its incidence appears to be rising, and the mortality caused by septic shock remains extraordinarily high [3]. Septic shock patients do not die from their infection per se but rather from multiple organ failure caused by an overwhelming host response. In fact, this essential mechanism has been implemented as a key part of the 2016 sepsis definition (SEPSIS-3) [1]. Despite tremendous efforts during the last decades, innovative approaches targeting this fundamental hallmark of the disease, thereby reducing organ dysfunction, are still lacking [4]. Undoubtedly, there is an unmet medical need to expand the current standard of care for these patients by a more specific intervention.

The investigators hypothesize that early therapeutic plasma exchange (TPE) in the most severely ill individuals with septic shock will dampen the maladaptive host response by removing injurious mediators thereby limiting organ dysfunction [5, 6]. Due to its non-selective nature in removing injurious mediators (virtually all pro-inflammatory cytokines, coagulatory molecules and growth factors including permeability mediators and glycocalyx sheddases, etc.), TPE might be more efficient than pharmacological blockade of single components of this process. Moreover, it might also reduce the molecular triggers of inflammation by removing pathogen associated molecular patterns (PAMPs) such as lipopolysaccharides and damage associated molecular patterns (DAMPs) that are released by pathogens or injured host cells.

A meta-analysis from 2014 found four single-center randomized controlled trials (RCTs) that analyzed TPE in sepsis. In adults, TPE was associated with a reduced mortality (relative risk 0.63) [7]. The largest of those trials showed a trend towards improved survival [8]. However, this study was underpowered and had included a heterogeneous group in terms of disease severity (e.g., less 60% with shock). Many of their patients might have recovered anyways with standard treatment. Of note, the American Society For Apheresis (ASFA) grades “sepsis with multi-organ dysfunction” as potential 2B, category III indication for TPE (= optimum role not established, decision should be individualized) [9]**.**

The investigators performed a single-center prospective pilot study to analyze safety, feasibility, and primary efficacy endpoints in preparation for the here proposed multicenter RCT in a septic shock cohort [10] with similar inclusion criteria (i.e., early and severe septic shock). TPE was found safe and feasible, and we observed positive effects with regard to secondary efficacy endpoints, e.g., rapid reduction of NE requirement [10]. A subsequentially conducted randomized controlled bi-center trial (Hannover and Bonn) in 20 vs 20 patients found a highly significant reduction in NE in the treatment group within 6 h compared to the SOC group (2). In 2020, Keith and co-workers additionally reported in a propensity score matched observational trial of septic shock patients treated with TPE a significantly better survival rate [11]. Incorporating these novel data from the last 2 years in a fixed effect meta-analysis, the investigators found a risk ratio for mortality of 1.49 [1.15; 1.94] favoring plasma exchange (unpublished data).

### Objective

As no adequately powered trial has been performed, the definite role of TPE as an adjunctive treatment option in septic shock remains unclear. Therefore, the objective of the here proposed study is to demonstrate that additive early TPE compared to SOC reduces the 28-day mortality in patients with septic shock.

### Trial design

**Fig. 1:**
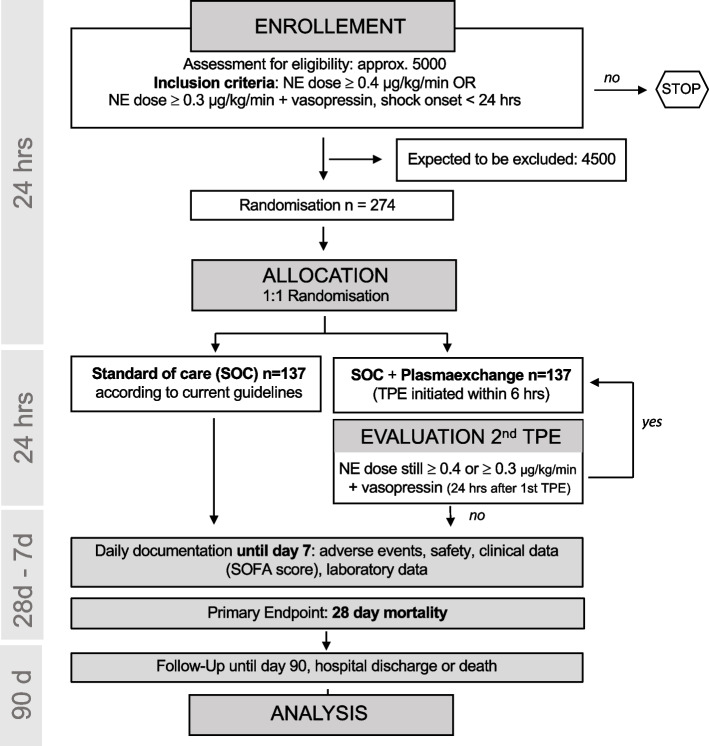
Trial structure

This is a randomized, prospective, multicenter, open-label, controlled, parallel-group interventional trial to test superiority of adjunctive TPE over standard of care treatment in patients with early septic shock. The study will have two arms: a treatment arm, in which patients receive a singular session of TPE using fresh frozen plasma (FFP) as replacement fluid (optional second TPE session after 24 h possible, see below) and a control arm, in which patients are treated with the current SOC (Fig. 1). The primary endpoint is 28 days mortality. Study participants will be followed up at 90 days and 1 year following study inclusion. This study is an investigator-initiated trial funded by the German Research Foundation (DFG, DA 1209/7–1).

## Methods

### Study setting

A total of 274 septic shock patients (1:1 randomization) will be included in approximately 26 study centers. The study is planned at intensive care units (ICUs) of the following medical facilities in Germany, Switzerland, and Austria (EXCHANGE-2 study group):

In Germany: Hannover Medical School, Medical Intensive Care, Hannover Medical School, Anesthesiology Intensive Care, University Hospital Bonn (UKB), University Hospital Charite, Hospital St. Joseph, Berlin, Hospital Braunschweig, Reinkenheide Hospital, Bremerhaven, University Hospital Erlangen, University Hospital Essen, University Hospital Hamburg Eppendorf, Hospital Hannover Region, University Hospital Halle, University Hospital Heidelberg, University Hospital Jena, University Hospital Kiel Schleswig–Holstein (UKSH), Kiel, University Hospital Cologne, Hospital Cologne-Meerheim, Hospital Magdeburg, University Hospital Muenster Anesthesiology, University Hospital Rostock, Marienhospital Stuttgart, Stuttgart, Germany, University Hospital Munich (TU), University Hospital Munich (TU)**,** University Hospital Zurich (Switzerland), Inselspital Bern (Switzerland), Landeskrankenhaus Innsbruck (Austria) and “Allgemeines Krankenhaus der Stadt Wien,” Vienna (Austria).

### Eligibility criteria

Patients have to fulfill all of the following inclusion criteria to be eligible for participation in the study:

1. Onset of septic shock within < 24 h (SEPSIS-3 definition) AND
2. Norepinephrine (NE) dose of ≥ 0.4 μg/kg/min ≥ 30 min OR
  ≥ 0.3 μg/kg/min + vasopressin (any dose)
  for ≥ 30 min (target mean arterial pressure (MAP) ≥ 65 mmHg) despite adequate
  crystalloid fluid resuscitation of ≥ 30 ml/kg body weight AND
3. Established vascular access suitable for plasma exchange independent of study inclusion (due to established indication of renal replacement therapy (RRT), expected need for RRT within the next 48 h or other medical reasons as assessed by treating physician team)

#### Patients are excluded from participation in the trial if they meet one of the exclusion criteria

1. Age < 18 years and > 80 years
2. Urogenital focus of infection
3. Heparin-induced thrombocytopenia (HIT)
4. Pregnancy
5. Known history of transfusion reactions
6. Vasopressor requirement due to non-septic shock (e.g. cardiogenic shock)
7. Ineligible for intensive care without restrictions or limitations

### Who will take informed consent

A stratified randomization approach will be used. Patients who meet the respective inclusion criteria and who or whose proxy or legal representative give written informed consent to participate will be included. If the legal representative is not available to give written consent at place, a telephone-based education of the legal representative will be performed and a consultant physician (not involved in the patient’s care) will sign the consent form on behalf. The written consent of the legal representative will then be obtained in retrospect as soon as possible. Once a patient meets the criteria of early (< 24 h) and severe septic shock (NE dose ≥ 0.4 μg/kg/min or NE dose of ≥ 0.3 μg/kg/min + vasopressin), the patient will be randomized. Permuted block randomization with variable block length and stratified by center, sex (m/f), pulmonary focus of infection (yes/no), and baseline (at screening) lactate (< 4.5/ ≥ 4.5 mmol/l) will be used to allocate patients to the intervention and the control groups in a 1:1 ratio. Randomization will be conducted centrally using a web-based approach (eCFR).

The investigator is responsible for obtaining patient’s/legal representative’s written informed consent after adequate explanation of the aim, study assessments, potential risks and benefits, and consequences of the study as well as alternative treatment options. If patients are incapable of giving consent due to unconsciousness, informed consent may be given by a legal representative who has been designated by the local court. After retrieval of capacity for informed consent, patients have to be informed about study-specific interventions that have already been done and about the treatments that are planned in the future.

The patient information/informed consent form and the consent form for acquiring biomaterial have to be signed in duplicate by the patient/legal representative and the investigator. One document will be given to the patient/legal representative, the other one remains in the trial investigator file (TIF) at the trial site. No study procedures are allowed to be conducted until patient’s/legal representative written informed consent has been obtained. The patient information/informed consent form has to be revised whenever important new information becomes available that may be relevant to the subject’s consent. The patients have to be informed and asked to give their consent to continue study participation by signing the updated form. Participation in this clinical trial is voluntary. Withdrawal from the trial at any time and for any reason is without any disadvantages to the patient’s further treatment. In order to enable an unbiased assessment of the primary endpoint, and given that informed consent from the patient/ legal representative is required in order for the patient to be included into the trial, the endpoint of mortality at 28 days will be obtained from all patients and analyzed in the form of an intention-to-treat analysis, indiscriminate of a withdrawal of consent at a later point. In case of a patient’s withdrawal of informed consent, all study data collected up to the point of consent withdrawal will be anonymized and employed for further analyses solely in its anonymized form. However, in cases for which data from formally withdrawn patients is of relevance for the security assessment of this trial, anonymization will be delayed and only performed at an unspecified later point in time when security clearance is given by the trial responsible.

### Interventions

#### Study procedure (control arm): standard of care treatment

Subjects enrolled in this study will be treated in accordance with evidence-based international guidelines for the management of sepsis [1, 12]. Explicitly, participation of a center in this study indicates conformity with the tenets of treatment outlined in the 2021 Surviving Sepsis Campaign [12]. Among other therapeutic strategies, this implies that basic hemodynamic targets such as a MAP > 65 mmHg, HR < 120 BPM, SaO_2_ > 94%, and Hb > 7 g/dl will be employed, and that norepinephrine and balanced crystalloids will be administered as the first vasopressor and fluid of choice, respectively. Use of dynamic preload parameters to guide fluid resuscitation is strongly encouraged. Furthermore, antibiotics will be administered according to local standards for as long as necessary indiscriminate of the duration of the study. Antibiotic stewardship, including daily assessment of antibiotic escalation and de-escalation, is strongly encouraged. Additionally, patients who conform to the definition of ARDS will be ventilated with a low tidal volume ventilation strategy (6 ml/kg IBW) and those with a moderate-to-severe ARDS will be positioned in prone position for more than 12 h daily. As recommended for patients in refractory septic shock, hydrocortisone 4 × 50 mg/day (or 200 mg/day as continuous infusion) should be given to all patients until shock resolution and for max. 7 days. No other experimental adjunctive therapies for septic shock, including immunoglobulins and/or hemoperfusion, will be permitted.

#### Study procedure (treatment arm): therapeutic plasma exchange (TPE)

The TPE treatment will be initiated within 6 h after randomization. Duration of TPE treatment is approximately 120–180 min. An additional second TPE can be performed at 24 h following randomization if the patient has responded to first treatment as indicated by any reduction of NE dose *and* if NE dose is still ≥ 0.4 µg/kg/min after 24 h. Both unfractionated heparin (UFH) and citrate may be used as anticoagulant medication. To ensure treatment comparability between different patients, we will replace plasma in a fixed ratio of 1.2 × the individual patient’s total plasma fluid. The individual patient’s total plasma fluid will be calculated on each trial site from hematocrit and body weight (BW) following the formula by Kaplan et al. (16). The formula used calculates the estimated plasma volume (ePV) as: *ePV* = *[[0.065* × *BW* [kg]*)]* × *[100-Hematocrit* [%]*]]* × *10* [ml]. To avoid potential allergic reactions, anti-histamines (Clemastin (Tavegil®) 2 mg and Ranitidin (Ranitic®) 50 mg, both given as intra-venous (i.v.) push), will be administered before TPE (and in the control group at time of randomization). No additional cortisone push should be given as anti-allergic prophylaxis immediately before TPE treatment.

### Outcomes

#### Primary endpoint

The primary endpoint is 28-day mortality (Table 1).

**Table 1:** Endpoints

PRIMARY Endpoint	28-day mortality
KEY SECONDARY Endpoint	
Extent of organ failure	indicated by: Mean Sequential Organ Failure Assessment (SOFA) score over the first 7 days following randomization and Organ support-free days (total days free of invasive ventilation, vasopressors/inotrops and RRT) until d28
(Further) SECONDARY Endpoints	
90-day mortality	
1-year mortality	
Length of stay (LOS)	Intensive Care Unit (ICU) LOS [days] Hospital LOS [days]
Basic hemodynamics 0 and 12 h, d1–7	Days free of vasopressor until day 28 [days] Norepinephrine dose [µg/kg/min] Dobutamine dose [µg/kg/min] Epinephrine dose [µg/kg/min] Vasopressin dose [U/kg/min] Vasoactive inotropic score (VIS) Mean arterial pressure (MAP) [mmHg] Heart rate (HR) [bpm] Central venous pressure (CVP) [mmHg] Central venous saturation (ScvO_2_) [%]
Extended hemodynamics 0 and 12 h, d1–7	Cardiac index (CI) [l/min/m^2^] Stroke volume variance (SVV) [%] Systemic vascular resistance index (SVRI) [dyn*s*cm-5*m^2^] Global end diastolic volume index (GEDI) [ml/m^2^] Extravascular lung water index (ELWI) [ml/kg] Pulmonary vascular permeability index (PVPI)
Arterial blood gas analysis (BGA) 0 and 12 h, d1–7	pH PCO_2_ [mmHg] HCO_3_^−^ [mmol/L] PO_2_ [mmHg] Lactate [mmol/L]
Respiratory function 0 and 12 h, d1–7	Days free of ventilator until day 28 [days] PO_2_/FiO_2_ ratio (PF ratio) Tidal volume (VT) [ml] Positive end-expiratory pressure (PEEP) [cmH_2_O] Peak pressure (Ppeak) [cmH_2_O] Plateau pressure (Pplat) [cmH_2_O] Respiratory Rate (RR) [1/min] Inspiratory Time (Tinsp) [s] Inspiratory Flow [l/min] End tidal CO_2_ (etCO_2_) [mmHg]
Renal function 0 and 12 h, d1–7, ICU discharge	Days free of renal replacement therapy (RRT) until day 28 [days] Presence of acute kidney injury (AKI) (KDIGO) [yes/no], 0 and 12 h, d1–7, ICU discharge AKI stage (KDIGO) [Stage 1 to 3], 0 and 12 h, d1–7, ICU discharge Need for RRT [yes/no], 0 and 12 h, d1–7, ICU discharge Estimated Creatinine-based Glomerular Filtration Rate (eGFR) (CKD-EPI) [ml/min], 0 and 12 h, d1–7, ICU discharge Fluid intake [ml/d], d1–7 Urine output [ml/d], d1–7 Ultrafiltration [ml/d], d1–7 Net daily fluid balance [ml/d], d1–7
Liver function 0 and 12 h, d1–7, ICU discharge	Bilirubin [µmol/l] Aspartate aminotransferase (AST) [U/l] Alanine aminotransferase (ALT) [U/l] Alkaline phosphatase (AP) [U/l] Gamma-glutamyl transferase (GGT) [U/l] Cholinesterase (CHE) [kU/l] Albumin [g/l]
Sepsis associated coagulopathy 0 and 12 h, d1–7	Differential full blood count (including hemoglobin (Hb) [g/dl], white blood cell count (WBC) [Tsd/µl], polymorphonuclear leucocytes (PMN) [Tsd/µl], lymphocytes [Tsd/µl], monocytes [Tsd/µl], platelets [Tsd/µl], and schistocytes [%]) Fibrinogen [g/l] D-Dimer [mg/l] International normalized ratio (INR) Lactate dehydrogenase (LDH) [U/l] Antithrombin (AT)-III [%] Protein C [%] vWF:Ag [IU/dl] (*only* at 0 and 24 h) ADAMTS13 [%] (*only* at 0 and 24 h) ISTH-DIC Score [0–8 points]
Inflammatory response 0 and 12 h, d1–7	C-reactive protein (CRP) [mg/l] Procalcitonin (PCT) [µg/l] Interleukin-6 (IL-6) [ng/ml] Ferritin [µg/l] Neutrophil/lymphocyte ratio
Cardiac function 0 and 12 h, d1–7, ICU discharge	Creatine kinase (CK) [U/L] Myoglobin [ug/L] NT-proBNP [ng/L] Troponin T, high sensitive [ng/L]
Secondary infections until ICU discharge	Presence [yes/no] Species [gram + /gram − /viral/fungal] Viral reactivation (HSV, EBV, and CMV copies by PCR) (*only* at days 7 and 14)
SAFETY ENDPOINTS	
Bleeding complications until day 7	Vascular access-related Intra cerebral bleeding Others
Bleeding complications until day 7	Acute myocardial infraction Thrombosis/ Embolism
Allergic reactions until day 7	Acute hemodynamic worsening + tachycardia
Infections until day 7	Infections related to the entry site of the dialysis catheter or new bloodstream infections
Transfusion associated lung injury (TRALI) until day 7	Significant worsening of respiratory function + typical X-ray deterioration
Severe thrombocytopenia until day 7	Occurrence of and time to severe thrombocytopenia (< 5000/μl)
Other device related Severe Adverse Events (SAEs) until day 7	Other device related SAEs such as air embolism or clot formation in the vein around the catheter with a risk of dislodgement of the clot to the lung
Other SAEs until day 7	SAEs that are not related to the critical illness / sepsis per se and are classified as possibly, probably or causally provoked by the intervention

#### Key secondary endpoint

The key secondary endpoint is the extent of organ failure (Table 1) indicated by both:

1. The mean Sequential Organ Failure Assessment (SOFA) score over the first 7 days following randomization with day 1 (d1) indicating the day of study inclusion (i.e., the day of the intervention). The SOFA score [13] will be recorded daily at inclusion and following the next 7 days.
2. Organ support-free days (total days free of invasive ventilation, vasopressors / inotrops and RRT) until day 28.

#### Secondary endpoints

As further secondary endpoints 90-day and 1-year mortality, intensive care unit length of stay (LOS), hospital LOS, (basic- and extended) hemodynamics, arterial blood gas analysis (BGA) parameters, respiratory-, renal- and liver function, sepsis associated coagulopathy, inflammatory response, cardiac function, and secondary infections will be analyzed (Table 1).

#### Safety endpoints

The following safety endpoints are assessed until day 7 following randomization (Table 1): bleeding complications, thrombotic complications, allergic reactions, infections related to the entry site of the dialysis catheter or new bloodstream infections, transfusion related lung injury, occurrence of, and time to severe thrombocytopenia (< 5.000/μl) and other device related serious adverse events (SAEs). Given the profound severity of disease in the patients under investigation (hospitalized patients under critical care, shock and (multi) organ failure) a variety of AEs and SAEs are expected to incept as part of the natural history of disease and will therefore not be reported. Furthermore, SAEs, that are not directly related to critical illness / sepsis per se and are classified as possibly, probably, or causally provoked by the intervention, will additionally be recorded until day 7. Potential SAEs that are directly related to critical illness will thus not be explicitly reported.

#### Exploratory endpoints

For exploratory scientific analyses, biomaterial (serum, plasma, urine, waste plasma from TPE) will be acquired longitudinally (at randomization, 12 and 24 h, as well as 3, 7, and 14 days following randomization) and multiple predefined scientific subprojects are planned.

### Participant timeline

A time schedule of enrolment, interventions, assessments, and visits for participants (SPIRIT Figure) is given as a schematic table (Table 2).

**Table 2:**
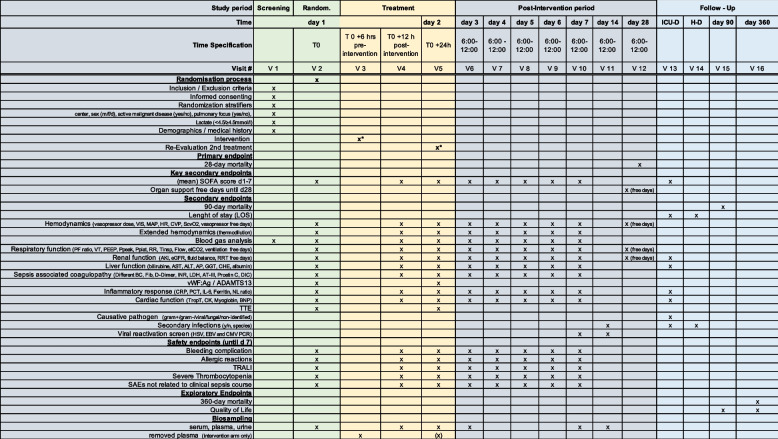
Schedule of Enrollment, Interventions and Assessments (SPIRIT Figure)

### Sample size

The primary aim of the study is to demonstrate superiority of SOC + TPE compared to SOC alone with regard to the primary endpoint 28-day mortality. In a prospective, monocentric, non-controlled pilot study of the TPE treatment in patients with septic shock, the observed 28-day mortality rate was 65%, while the predicted mortality rate using the APACHE-II-score was as high as 91% [10]. Meanwhile, two randomized clinical trials have been conducted. In one (small, *n* = 20 vs 20) randomized control trial, the observed mortality rate of the TPE-treated patients was 40% in contrast to 50% in the control group, resulting in a risk difference of − 10% with a 95% CI of [− 0,41;0,21] [14]. In another randomized control trial, the observed mortality rate of the treated patients was 33.3 and 53.8% in the control group, resulting in a risk difference of − 21% with a 95% CI of [− 0.39; − 0.02] [8]. For sample size calculation, we considered the two RCTs only and combined the outcome in a meta-analysis. The treatment procedures were similar, and the two trials are very homogeneous (I2 of 0%). The meta-analysis resulted in a risk difference of − 18% with a 95% CI of [− 0.34; − 0.02]. This risk difference was used in the sample size calculation. Sample size calculation was done for a two-group continuity corrected chi-square test (Query Advisor 8.7.1.0). To detect a reduction in 28-day mortality rate from 60 to 42% in the experimental group with a two-sided type I error rate of 5% and a power of 80%, at least 131 patients per group are required. Thus, the total sample size to detect the expected effect on the 28-day mortality is 262 patients. Stratification for sex, center, pulmonary focus of infection (yes/no), and lactate (< / ≥ 4.5 mmol/) at randomization is assumed to increase power of the statistical test. Considering a drop-out rate of 5%, a total of 274 patients will be assigned to the trial, i.e., recruited.

### Recruitment

The EXCHANGE-2 study group will secure adequate recruitment and conduction of the trial. Every week during the trial period, the recruitment numbers will be assessed, monitored by the Hannover Clinical Trails Center (Zentrum für Klinische Studien (ZKS)) and will be reported to the principal investigator. Patients under investigation are critically ill and therefore drop-out or loss to follow-up or other intercurrent events are unlikely to hinder the observation of the primary endpoint.

### Assignment of interventions: allocation

Permuted block randomization with variable block length stratified by sex, center, pulmonary focus of infection (yes/no), and lactate (< / ≥ 4.5 mmol/) at randomization will be used to allocate patients to both study arms. Only the statistician and the unblinded data manager will have access to the randomization list. The allocation is performed via the eCRF system. Only after the patient has been documented with in- and exclusion criteria in the eCRF by the study site, the allocation of the patient to a treatment group will be displayed in the eCRF automatically according to the sequence determined by the randomization list. For the study sites, it is not possible to see the randomization list.

### Blinding

The clinical team responsible for the participant (physicians, nurses, and others) involved with direct patient care will not be blinded to allocation group due to the inherent difficulty in blinding the intervention. Measures will be taken to ensure that the information about allocation will not disseminate beyond the immediate group of caregivers responsible for patient care. The intensive care physician will not be allowed to share any information regarding the allocation group. Patients, their legal representatives, and family will only be informed that the patient has been randomized into the study. Health personnel responsible for outcome assessment at follow-up will be blinded to the allocation of the intervention when possible.

The author group, trial statistician, and the trial coordinating team will be blinded to group allocation. In order to enable an unbiased assessment of the intervention and manuscript writing process, the two study arms will be coded as “X” and “Y”. Two manuscripts will be drafted one assuming that “X” corresponds to the TPE group and “Y” to the control group—and one draft assuming the contrary. The code will be broken upon acceptance of both drafts by the author group.

### Data collection and management

#### Data management

All study data will be collected by the investigator and/or other study personnel. An online clinical trial data base is provided, in which the data are entered via an electronic Case Report File (eCRF). Authorized and trained staff of the study sites will enter the data in the eCRF. SAEs will additionally be documented on paper forms. Verification of the data in the eCRF occurs by monitoring as well as via range, validity, and consistency checks programmed in the system. Additionally, manual queries can be raised in the system by authorized study staff if further discrepancies are detected. Based on the queries, the investigator can review and answer the found discrepancies directly in the system. All changes of data entered in the eCRF can be followed by an audit trail. A quality control will be performed before the database is closed. This procedure is documented. Finally, data transfer takes place for statistical evaluation.

#### Data protection

All study staff has to give due consideration to data protection and medical confidentiality. The collection, transfer, storage, and analysis of personal study-related data are performed pseudonymized according to national regulations. This trial will conform in its entirety with the Regulation (EU) 2016/679 of the European Parliament and of the Council of 27 April 2016 on the protection of natural persons with regard to the processing of personal data and on the free movement of such data. The declaration of data protection is contained within the patient information/informed consent form.

#### Data safety and monitoring board

An independent Data Safety Monitoring Board (DSMB) will be implemented to detect possible harms and to assure continuous risk/benefit assessment. The DSMB is a group of independent experts external to the clinical investigation assessing the progress, safety data, and if needed, critical efficacy endpoints. The members of the DSMB are the following: Prof. Dr. Frank Brunkhorst, UK Jena, Zentrum für klinische Studien & Center for Sepsis Care and Control; Prof. Dr. Johannes Oldenburg, UK Bonn, Institut für Experimentelle Hämatologie und Transfusionsmedizin; Prof. Dr. Reinhard Klingel, Apherese Forschungsinstitut, Köln, Universitätsmedizin Mainz; Prof. Dr André Scherag, UK Jena, Institut für Biometrie, Co-Speaker of the Center for Sepsis Control and Care (CSCC).

### Collection and storage of biomaterial 

Blood samples of approx. < 40 ml (2 × serum, 2 × plasma, 2 × citrate, 2 × hirudin S-Monovette for immunostimulation assay, and 1 × Streck – Cyto-Chex BCT tube for immunophenotyping) and urine samples of approx. 15 ml (2 × urine) will be obtained at Visit 2 (at randomization, BIO-1), at visit 4 (12 h after randomization, BIO-2), at visit 5 (24 h after randomization, BIO-3), at visit 6 (48 h after randomization, BIO-4), at visit 10 (day 7, BIO-5), and visit 11 (day 14, BIO-6) for the evaluation of the planned exploratory endpoints. Additionally, 20 ml of the removed plasma will be collected (in the treatment group only) at visit 3 (TPE-1) and (if a second TPE is performed) at visit 5 (TPE-2). Details regarding the collection, processing, storage, and shipment of samples will be included in the lab manual.

### Statistical methods

#### Analyses populations

The intention-to-treat (ITT) population comprises all randomized patients. Patients will be analyzed as randomized independently of the performed intervention. Primary analyses of all efficacy endpoints will be conducted on the ITT population.

The per-protocol (PP) population comprises all patients that received the randomized intervention and were complying with the study protocol until the end of the follow-up period. Supplementary efficacy analyses will be conducted on the PP population.

The safety population comprises all patients that received the study intervention. Patients will be analyzed as treated. Analyses of safety endpoints will be conducted on the safety population.

#### Analysis of the primary endpoint

The primary endpoint 28-day mortality (dead at 28 days: yes or no) which will be analyzed using a logistic regression model adjusting the treatment effect for sex, pulmonary focus of infection (yes/no), baseline lactate (< 4.5/ ≥ 4.5 mmol/l), and center will be used. Superiority of the experimental intervention compared to the control intervention can be concluded if the upper boundary of the two-sided 95% confidence interval for the adjusted odds ratio (experimental intervention / control intervention) is below 1.

Patients will be followed up even after premature discontinuation of study intervention, and all collected data will be used in the primary analysis. Since patients enrolled in this study are critically ill, the rate of drop-out and loss to follow-up is expected to be very low. Nevertheless, if the vital status at day 28 is unknown for a patient, this patient will be considered dead at day 28 in the primary analysis.

As a sensitivity analysis, the missing vital status at day 28 will be handled using the multiple imputation.

#### Analyses of secondary endpoints

Analyses of the key secondary endpoints will be performed as follows:

(i) The mean SOFA score will be analyzed as degree of organ failure measured by the per-patient mean daily SOFA score over 7 days. In case a patient dies before day 7, the daily SOFA score will be registered as 24 points for the following days until day 7. A linear regression model will be used for comparing the treatment arms, which includes mean daily SOFA score over 7 days as dependent variable, and treatment group, sex, pulmonary focus of infection (yes/no), baseline lactate (< 4.5/ ≥ 4.5 mmol/l), and center as independent variables. Superiority of the experimental intervention compared to the control intervention can be concluded if the upper boundary of the two-sided 95% confidence interval for the adjusted mean difference (experimental intervention minus control intervention) is below 0.
  A sensitivity analysis for the SOFA score will be performed using an exact test based on trimmed means [15]. This method follows the ITT principle in taking all randomized patients into account in the analysis without hypothesizing SOFA values for patients who die before day 7.
(ii) Organ support-free days until day 28, determined as total days without need for invasive ventilation, vasopressor/inotropes, and RRT until day 28, will be analyzed similarly with a linear regression model with stratification factors of the randomization as co-variates. In case a patient dies before day 28, each remaining day until day 28 will be counted as a day with organ failure.

The confirmatory assessment of key secondary endpoints will be done in the above specified order as soon as the primary analysis of the primary endpoint is significant.

Other secondary endpoints:

The 90-day mortality (dead at day 90: yes or no) will be analyzed in line with the primary analysis of 28-day mortality. In addition, 28-/90-day mortality will be analyzed as time-to-event endpoint. The survival curves until day 28/90 will be estimated using the Kaplan–Meier method and compared using the log-rank test. Additionally, a Cox regression model will be utilized to estimate the hazard ratio between the treatment arms.

Other secondary endpoints (e.g., ICU length of stay, hospital length of stay, hemodynamics, arterial blood gas analysis) will be summarized by treatment group and compared with appropriate statistical tests.

#### Interim analyses

No interim analyses will be performed.

#### Analyses of safety endpoints

Absolute and relative frequencies of all safety endpoint events will be displayed for the whole population and separately for experimental and control group and will be compared using chi-squared tests.

#### Subgroup analyses

Subgroup analyses will be performed for the primary and key secondary endpoints in the relevant subgroups including sex, age (≥ 60/ < 60), pulmonary focus of infection (yes/no), RRT (yes/no), pre-existing congestive heart failure NYHA III/IV (yes/no), pathogen (gram + /gram −), baseline lactate concentration (< 4.5/ ≥ 4.5 mmol/l).

### Oversight and monitoring

#### Responsibilities

This study will be conducted in compliance with the ICH GCP guidelines (as far as possible for this kind of study) and the Declaration of Helsinki. Investigators must have sufficient time to conduct the clinical study in compliance with the study protocol. Furthermore, they have to accurately and completely enter study data in the eCRF. Investigators are responsible for obtaining informed consent of the patients as well as for the preparation and maintenance of adequate case files in order to record observations and other data relevant for this clinical study. Besides, they have to file the study-related records in the ISF and have to maintain its actuality. They will permit study-related monitoring visits. The investigator must provide direct access to the study site’s facilities, to source documents, and to all other study documents.

#### Favorable opinion of independent ethics committee

Study protocol, patient information with consent, and substantial amendments will be assessed by the responsible ethics committees. Favorable opinion of the specific Independent Ethics Committee (IEC) must be available prior to study start at all study sites. The ethical committee of MHH has initially approved the protocol as lead ethical institute in 2015 (No.2786–2015) and has renewed its approval in 2020 (No.8852_MPG_23b_2020) and most recently in 2023 (No. 10607_BO_SK_2022).

#### Monitoring

Monitoring is performed for reasons of quality assurance and to verify that the study is conducted according to the protocol as well as to legal and regulatory requirements applicable for clinical trials. Quality assurance will be based on three components: on-site monitoring, central monitoring, and extensive training. All trial-related processes will follow the SOPs of the Hannover Clinical Trials Center (Zentrum für Klinische Studien (ZKS)). Key documents and processes are subject to internal review, following the SOPs of the ZKS. Central monitoring will include a timely query management process based on consistency and plausibility checks automatically generated from the database, combined with a reminder process for missing documentation. A total of 3 on-site monitoring visits (1 visit/year) will be performed by monitors of the ZKS. Pre-study visits will be performed in each recruiting center via phone by ZKS-independent monitors to instruct the local investigators in how to follow the study protocol and documentation of data. An on-site initiation visit has to be performed, before a site is allowed to start recruitment to ensure adherence with all study procedures by the monitor of ZKS. Periodic monitoring visits and source data verification will be done according to a risk-adapted approach and to assure high data quality and patient safety and to check informed consents. The focus of on-site monitoring will be on the verification of informed consent documents, eligibility criteria, primary endpoint, key secondary endpoints, and safety aspects (100% of the patients included). One hundred percent source data verification will be performed for the first patient of each trial site and for 20% of all further patients, respectively. Close out visits will be done at the end of the trial and in case a site will be closed.

#### Record retention

All relevant study-related documents have to be archived for at least 10 years after completion or premature discontinuation of the clinical study. The investigator agrees to keep the ISF, including the identity of all participating patients, all original signed informed consent forms, detailed records of treatment, all other applicable study-related documents, and source documents. The records should be retained by the investigator for at least 10 years after completion or premature discontinuation of the clinical study. Source data have to be kept according to national regulations.

#### Insurance

The trial will be covered by the trial site`s individual liability insurance (Haftpflichtversicherung). All subjects/legal representatives will be informed about their rights and obligations in regard to insurance policies before participating in the study. A copy of the insurance policies will be handed out to each patient/legal representative.

#### Financing

This study is funded by the German Research Foundation (DFG, DA 1209/7–1).

#### Amendments

Each amendment of essential study documents has to be approved by the study initiators. Favorable opinion of IEC is required for amendments prior to implementation.

#### Dissemination plans

It is anticipated to publish the results of the clinical trial in a scientific medical journal and at national and international meetings. The responsible investigator will designate the first and the last authors of the publication. The order of subsequent authors will be allocated according to the number of patients recruited by each site. The trial was registered at ClinicalTrials.gov (NCT05726825, Registered on 02/14/2023). Data will be available to investigators upon reasonable request.

## Discussion

To the present time, no adequately powered randomized clinical trial has investigated the effect of TPE on survival in septic shock patients. Previous studies investigating TPE in sepsis have either been underpowered or included a heterogenous study population [7, 8]. Therefore, despite promising data demonstrating more rapid hemodynamic stabilization [10, 14], the role of TPE as a potential adjunctive treatment option in septic shock remains unclear. As we have observed the most obvious clinical effects (during the feasibility trial) in sepsis patients that have been treated very early after onset of shock and that demonstrated particularly profound hemodynamic instability, we have designed the inclusion criteria of the here described study accordingly. This effort to homogenize the participants (early and severe septic shock) stands in contrast to previous trials that have investigated a broader range of septic patients.

TPE using human plasma as replacement fluid has potential adverse effects, including infectious and non-infectious (allergic reaction, transfusion associated lung injury (TRALI), citrate toxicity, hypotension) [16] with pruritus and urticaria most commonly observed [17]. However, severe adverse events are extremely rare [18] and incidence of adverse events requiring discontinuation of treatment lies at around 0.2% [17]. Of note, no adverse events were observed in our previous EXCHANGE feasibility pilot study [10] and the randomized EXCHANGE-1 trial, investigating patients with literally the same characteristics [19]. To avoid potential allergic reactions, anti-histamines will be administered before TPE (and in the control group at time of randomization). Importantly, there is no additional invasive exposure of the patient. The central line used for TPE will be inserted for clinical management (e.g., RRT) only and all biosamples will be drawn via catheters (central venous and arterial), which have been placed independent of biosampling for treatment and monitoring indication. Therefore, no additional puncture of the skin needs to be performed for biosampling.

The investigators convinced that a risk–benefit consideration clearly favors performance of the here proposed clinical trial. Importantly, this is an investigator-initiated trial, funded by the DFG without any competing commercial or financial interests involved.

Furthermore, accompanying systematic and longitudinal biosampling together with predefined innovative center-specific research projects will most certainly enable highly stimulating scientific investigations in the field of septic shock research.

## Conclusions

This trial has substantial clinical relevance as it evaluates a promising adjunctive treatment option in septic shock patients suffering from an extraordinary high mortality. A positive trial result could change the current standard of care in severe septic shock.

## Data Availability

Data will be available to investigators upon reasonable request.

## Abbreviations

AE: Adverse event
AKI: Acute kidney injury
ALT: Aspartate aminotransferase
ARDS: Adult respiratory distress syndrome
ASFA: American Society for Apheresis
AST: Aspartate aminotransferase
AT-III: Antithrombin-III
BGA: Blood gas analysis
BMI: Body Mass Index
CHE: Cholinesterase
CI: Cardiac index
CI: Confidence Interval
CK: Creatine kinase
CRA: Clinical Research Associate
CRP: C-reactive protein
CVP: Central venous pressure
CTS: Clinical Trial Services
DAMP: Damage associated molecular pattern
DFG: German Research Association
DIC: Disseminated intravascular coagulation
DSMB: Data Safety Monitoring Board
eCRF: Electronic case report form
eGFR: Estimated glomerular filtration rate
ELWI: Extravascular lung water index
ePV: Estimated plasma volume
ET: Early termination
etCO_2_: End tidal CO_2_
FFP: Fresh frozen plasma
FiO_2_: Fraction of inspired oxygen
GCP: Good Clinical Practice
GCS: Glasgow Coma Scale
GEDI: Global end diastolic volume index
GGT: Gamma-glutamyl transferase
Hb: Hemoglobin
HIT: Heparin-induced thrombocytopenia
HR: Hazard ratio
HR: Heart rate
IB: Investigator’s Brochure
ICF: Informed Consent File
ICH: International Conference on Harmonization
ICU: Intensive care unit
IEC: Independent Ethics Committee
IL-6: Interleukine-6
INR: International normalized ratio
ISF: Investigator Site File
ISTH: International Society on Thrombosis and Hemostasis
ITT: Intention-to-treat
i.v.: Intra-venous
KDIGO: Kidney Disease Improving Global Outcomes
LDH: Lactate dehydrogenase
LOS: Length of stay
LV-EF: Left ventricular ejection fraction
MAP: Mean arterial pressure
MHH: Hannover Medical School
NE: Norepinephrine
PAMP: Pathogen associated molecular pattern
PaCO_2_: Partial pressure of carbon dioxide
PaO_2_: Partial pressure of oxygen
PCT: Procalcitonin
PD: Protocol deviation
PEEP: Positive end-expiratory pressure
PP: Per protocol
Ppeak: Peak pressure
Pplat: Plateau pressure
POC: Point of care
PVPI: Pulmonary vascular permeability index
RCT: Randomized controlled trial
RR: Respiratory rate
RRT: Renal replacement therapy
RVEF: Right ventricular ejection fraction
SAE: Serious adverse event
SCR: Screening
ScvO2: Central venous saturation
SOC: Standard of Care
SOFA: Sequential organ failure assessment score
SOP: Standard Operating Procedure
SVRI: Systemic vascular resistance index
SVV: Stroke volume variance
TIF: Trial investigator file
TPE: Therapeutic plasma exchange
TRALI: Transfusion associated lung injury
TTE: Transthoracic echocardiography
TU: University Hospital Munich
UK: University Hospital Cologne
UKB: University Hospital Bonn
UKE: University Hospital Hamburg Eppendorf
UKH: University Hospital Heidelberg
UKJ: University Hospital Jena
ULN: Upper limit of normal
UN: Unscheduled visit
USZ: University Hospital Zurich
VEGF: Vascular endothelial growth factor
VIS: Vasoactive inotropic score
VT: Tidal volume
WBC: White blood cell count
ZKS: (Hannover) Clinical Trails Center (Zentrum für Klinische Studien)
